# Long-Term Effectiveness of a Monofocal Intraocular Lens (IOL) Enhanced for Intermediate Vision: A 5-Year Follow-Up Study

**DOI:** 10.3390/jcm14165831

**Published:** 2025-08-18

**Authors:** Rita Mencucci, Giovanni Romualdi, Alberto Carnicci, Fabio Panini, Matilde Buzzi, Fabrizio Giansanti

**Affiliations:** 1 Eye Clinic, Department of Neuroscience, Psychology, Pharmacology and Child Health (NEUROFARBA), University of Florence, Largo Brambilla 3, 50134 Florence, Italy; 2 Unit of Ophthalmology, Department of Medicine, Surgery and Neuroscience, University of Siena, Viale Mario Bracci 16, 53100 Siena, Italy

**Keywords:** enhanced monofocal IOL, Tecnis Eyhance, intermediate vision, long-term outcomes, cataract surgery, defocus curve, contrast sensitivity, posterior capsule opacification, glare, spectacle independence

## Abstract

**Background/Objectives**: The Tecnis Eyhance is an enhanced monofocal intraocular lens (IOL) designed to improve intermediate vision without compromising distance clarity or increasing the incidence of photic phenomena. Although short-term results have been encouraging, long-term data remain limited. This study presents the 5-year follow-up of a previously published 6-month clinical evaluation, aiming to assess the stability of visual, optical, and patient-reported outcomes over time. **Methods**: A single-center retrospective study of 18 patients (36 eyes) undergoing bilateral Tecnis Eyhance IOL implantation was conducted. The same cohort from the original 6-month study was re-evaluated after a mean follow-up of 5 years. Visual acuity (distance, intermediate, near), defocus curves, contrast sensitivity, optical quality, effective lens position (ELP), halo size, and patient-reported measures were assessed. **Results**: Visual acuity remained stable across all distances, with binocular uncorrected intermediate visual acuity (UIVA) ≤ 0.2 logMAR in all patients. No significant changes were observed in optical quality parameters or contrast sensitivity. ELP remained consistent over time (*p* = 0.298), and posterior capsule opacification (PCO) requiring Nd:YAG capsulotomy developed in 5% of the eyes. Halo size was mild, and subjective glare perception did not increase. Spectacle independence remained high for distance (100%) and intermediate (more than 75%) tasks. **Conclusions**: This 5-year follow-up study confirms the long-term stability and effectiveness of the Tecnis Eyhance IOL. These findings support its long-term use as a stable monofocal IOL with enhanced intermediate function.

## 1. Introduction

Cataract surgery is increasingly regarded as a refractive procedure, a trend that is expected to accelerate in the coming years. Patients now have higher expectations for visual quality and functional vision, seeking greater independence from spectacles. Although multifocal intraocular lenses (IOLs) offer the potential for glasses-free vision at multiple distances, monofocal IOLs remain the most widely implanted due to their lower incidence of photic phenomena, such as glare and halos, which are more prevalent in multifocal designs owing to their refractive or diffractive optical structures. However, conventional monofocal IOLs, which provide a single focal point optimized for distance vision, typically do not address the intermediate vision needs required for many daily activities, including computer use, playing musical instruments, driving, and navigating uneven surfaces. In recent years, the demand for IOLs capable of providing both distance and intermediate vision has increased. This shift is partly driven by the decreasing average age of cataract surgery patients and their evolving visual demands. Many individuals over the age of 60 now engage in activities that require clear intermediate vision, often in low-lighting conditions.

At the same time, the availability of intraocular lenses (IOLs) designed to provide an extended range of vision has grown significantly. This trend reflects a growing emphasis on enhancing intermediate visual performance while minimizing the optical side effects often associated with multifocal IOLs. To address this, Extended Depth of Focus (EDOF) IOLs were developed to improve intermediate vision with reduced photic disturbances, such as glare and halos.

More recently, a new category known as “enhanced monofocal IOLs” has emerged. These lenses offer the same high-quality distance vision as traditional monofocal lenses but with improved intermediate visual performance. Given the increasing diversity of IOL designs, establishing a clear classification system has become more complex. To address this need, a novel evidence-based functional classification was recently proposed, grouping IOLs based on the range of field (RoF) provided by their defocus curves [1]. This system categorizes IOLs into two primary types: PARTIAL-RoF and FULL-RoF. Within the PARTIAL-RoF category, three subgroups are defined based on the extent of intermediate vision achieved: narrow, enhanced, and extended. In contrast, the FULL-RoF category includes IOLs that differ in their visual acuity (VA) profiles from intermediate to near distances, with subtypes characterized as continuous, smooth transition, or steep transition, depending on the gradient of focus improvement.

The Tecnis Eyhance (ICB00) (Johnson & Johnson Vision, Irvine, CA, USA), introduced in 2019, was the first enhanced monofocal IOL designed to extend depth of focus while preserving the benefits of standard monofocal lenses. Unlike multifocal designs, the ICB00 features a ring-free refractive optic with a power profile that gradually increases from the periphery to the center, creating a unique high-order aspheric anterior surface. This design aims to improve intermediate vision without compromising distance clarity. Early studies have shown that the Tecnis Eyhance IOL delivers superior intermediate vision compared to conventional monofocal IOLs, with no compromise in distance vision or contrast sensitivity [2]. Despite these promising short-term results, long-term data on visual performance, optical stability, and patient satisfaction with the Tecnis Eyhance IOL remain limited. Assessing these parameters over extended follow-up is essential to confirm the durability of the lens’s benefits and to better inform clinical decision-making.

The present study reports on the 5-year postoperative outcomes of a cohort previously evaluated at 6 months following bilateral Tecnis Eyhance IOL implantation. This long-term follow-up provides valuable insights into the sustained performance and real-world benefits of the Tecnis Eyhance IOL.

## 2. Materials and Methods

### 2.1. Study Design and Patients

A single-center, retrospective review of consecutive patients who underwent bilateral Tecnis Eyhance IOL implantation between January and September 2019 was conducted. The cohort of patients evaluated at the 5-year follow-up was the same as that reported by Mencucci et al. in the previously published 6-month postoperative study [3]. All patients were fully informed about the study protocol and provided written informed consent prior to participation. The study was conducted at the Eye Clinic, Department of NEUROFARBA, University of Florence, Italy, in accordance with the tenets of the Declaration of Helsinki and with the approval of the institutional review board (Protocol number FA000459, ID study CEAVC24175).

As previously reported [3], the exclusion criteria included corneal astigmatism of more than 0.75 diopters (D), amblyopia, axial length exceeding 25.0 mm, previous ocular surgeries (including corneal or refractive procedures), chronic or recurrent uveitis, active ocular disease or external/internal infections, diabetes mellitus with retinal involvement, glaucoma or intraocular pressure ≥ 24 mm Hg, pseudoexfoliation syndrome, pathological miosis, and use of alpha-blockers associated with intraoperative floppy iris syndrome. Additional exclusion criteria were a history of choroidal hemorrhage, keratoconus, corneal endothelial dystrophy, and an education level below secondary school.

For postoperative evaluations, only patients without posterior capsule opacification (PCO) or those who had been successfully treated with Nd:YAG laser capsulotomy were included.

### 2.2. Intraocular Lens

The Tecnis Eyhance IOL (ICB00) (Johnson & Johnson Vision, Irvine, CA, USA) is a single-piece 6.0 mm hydrophobic acrylic lens with a modified aspheric anterior optic. This lens has a thickness deviation of 1.5 µm with a diameter of about 2 mm in the center and a continuous power profile that increases gradually from the periphery toward the center, extending the depth of focus and improving intermediate vision compared with conventional monofocal IOLs [4]. The Tecnis Eyhance IOL employs a purely refractive design, free of diffractive rings or zonal transitions. The absence of zones, discontinuities, and diffractive structures is expected to maintain straylight and photic phenomena at the same level as a standard aspheric monofocal IOL. The lens is designed to provide the same degree of primary spherical aberration correction for a 6-mm aperture (−0.27 µm) as the Tecnis family of IOLs (Johnson & Johnson Vision, Irvine, CA, USA) [5].

The IOL power and the predicted postoperative refractive outcome were based on biometric measurements from the IOLMaster 500 device (Carl Zeiss Meditec AG, Jena, Germany). Calculations were performed using different IOL power calculation formulas depending on axial length: the Holladay 1 formula was applied for eyes with axial lengths between 22.0 mm and 25.0 mm, while the Hoffer Q formula was used for axial lengths ≤ 22.0 mm [6,7]. The selected IOL power aimed to achieve a postoperative refraction as close to emmetropia as possible.

### 2.3. Surgical Technique

As previously described by Mencucci et al. [3], all procedures were performed by a single experienced surgeon (R.M.) using either topical or peribulbar anesthesia. A temporal clear corneal tunnel incision was created, followed by a capsulorhexis approximately 5.5 mm in diameter. Phacoemulsification was performed using the stop-and-chop technique with the Centurion Vision System (Alcon, Fort Worth, TX, USA). At the conclusion of the procedure, intracameral cefuroxime (Aprokam^®^, Thea Pharmaceuticals, Clermont-Ferrand, France) was administered into the anterior chamber, and corneal incisions were sealed with hydrosutures. Postoperative management included topical antibiotic and anti-inflammatory prophylaxis, as well as a course of nonsteroidal anti-inflammatory drugs (NSAIDs) for one month. Patients were also prescribed a combined corticosteroid–antibiotic eye drop regimen, administered four times daily for the first four days and three times daily for the subsequent seven days.

### 2.4. Postoperative Evaluation

Five-year postoperative evaluations were conducted using a protocol consistent with that employed in the study by Mencucci et al. [3]. Assessment included slit-lamp biomicroscopy, tonometry, and both subjective and objective refraction. Under photopic conditions, monocular and binocular uncorrected distance visual acuity (UDVA), uncorrected intermediate visual acuity (UIVA at 66 cm), uncorrected near visual acuity (UNVA at 40 cm), corrected distance visual acuity (CDVA), distance-corrected intermediate visual acuity (DCIVA), and distance-corrected near visual acuity (DCNVA) were recorded. Spherical addition required for corrected intermediate visual acuity (CIVA) and corrected near visual acuity (CNVA) was assessed both monocularly and binocularly. Additional tests included binocular defocus curves, contrast sensitivity, optical quality, halometry, patient-reported satisfaction questionnaires, and effective lens position (ELP) evaluation.

Distance visual acuity (UDVA and CDVA) was recorded at 4 m using a high-contrast (96%) ETDRS chart housed in an illumination cabinet equipped with an 85 cd/m^2^ lamp filter (Precision Vision). Intermediate and near visual acuities were assessed using high-contrast ETDRS printed charts (Precision Vision) under photopic lighting conditions, standardized by adjusting a halogen lamp’s potentiometer according to real-time room illumination measured by a calibrated light meter (ST-1300, STANDARD Instruments Co., Ltd., Hong Kong, China). To avoid memorization bias, different ETDRS charts were randomly assigned at each visit.

Binocular defocus curves were obtained with the patient’s best distance correction in place. Defocus was induced by adding trial lenses in 0.50 diopter (D) increments over a range from +1.00 D to −2.50 D, measuring visual acuity at each step.

Contrast sensitivity was measured binocularly under photopic conditions (85 cd/m^2^) using the Functional Acuity Contrast Test (FACT) chart in the Optec 6500 Vision Tester (Stereo Optical Co., Chicago, IL, USA).

Objective optical quality was evaluated using the AcuTarget HD Analyzer (Visiometrics USA, Costa Mesa, CA, USA), an Optical Quality Assessment System (OQAS) based on double-pass wavefront technology. Measurements were taken with a 4.0 mm pupil and included the objective scatter index (OSI), modulation transfer function (MTF) cutoff, and point spread function (PSF), expressed as the Strehl ratio. The OSI quantifies intraocular light scatter by comparing the peripheral and central light distribution within the double-pass image. The MTF cutoff indicates the highest spatial frequency at which contrast is maintained, while the PSF reflects the retinal image sharpness. The Strehl ratio, calculated as the ratio of the measured PSF to the ideal diffraction-limited PSF, provides an indicator of overall optical system quality, where a value of 1 denotes a theoretically perfect optical system [8].

Halometry, performed exclusively at the 5-year follow-up (as the device was unavailable during the 6-month evaluation), assessed the presence of lens-induced halos [9]. In a dark room, patients were seated 2 m from an LED source located at the center of an iPad (Apple Inc., Cupertino, CA, USA), controlled via Bluetooth from an iPhone (Apple Inc., Cupertino, CA, USA). Letters sized 0.3 LogMAR were projected proceeding from the periphery toward the center of the iPad (Aston App Halometer, Aston EyeTech Ltd., Birmingham, UK), where the light source is located, until recognized by the patient. This evaluation is repeated in each of the 8 or 6 directions (at the operator’s discretion) to determine the objective area of obscuration caused by the patient’s halo. For each patient, a halometer glare map was created to represent the halo area.

Subjective perception of photic phenomena (glare and halos) was assessed using items 17 and 38 from the glare subscale of the National Eye Institute-Refractive Error Quality of Life-42 (NEI-RQL-42) questionnaire [10]. Spectacle independence was evaluated using the Patient-Reported Spectacle Independence Questionnaire (PRSIQ) [11]. Before completing the questionnaires, the investigator provided standardized explanations and examples of visual tasks at various distances. Patients then completed the forms independently.

Moreover, to evaluate the long-term IOL stability, effective lens position (ELP) was assessed at 6 months and 5 years postoperatively using AS-OCT with the MS-39 device (Costruzione Strumenti Oftalmici, Florence, Italy) in a single-line scan mode. The ELP was measured using an image chamber tool, defined as the distance from the central corneal endothelium to the anterior surface of the IOL. Patients were seated and instructed to fixate on the internal target. Two images were captured at 0 degrees and 90 degrees, and the highest quality image was selected for analysis. No topical cycloplegic agents were administered during the exams, and all scans were performed by the same examiner.

### 2.5. Statistical Analysis

Statistical analysis was performed using IBM SPSS Statistics for Windows (version 30.0, IBM Corp.) and Stata software (version 19.0, StataCorp). The Shapiro–Wilk test was used to assess the normality of data distributions. For continuous variables (e.g., visual acuity, effective lens position, contrast sensitivity, optical quality parameters), comparisons between the 6-month and 5-year follow-up were conducted using the paired *t*-test for normally distributed data or the Wilcoxon signed-rank test for non-normally distributed data. Only cases with complete data available at both time points were included in the paired statistical analyses, while those lost to follow-up were excluded from these comparisons. A *p*-value of less than 0.05 was considered statistically significant. Results are presented as mean ± standard deviation (SD) for continuous variables and as frequency (percentage) for categorical variables.

## 3. Results

A total of 20 patients were included in the Tecnis Eyhance 6-month group, while 18 patients were included in the Tecnis Eyhance 5-year group, with a mean follow-up of 5.6 ± 0.4 years. Only two patients were excluded from the 5-year follow-up due to the development of age-related macular degeneration (AMD) in one eye. Their baseline characteristics and 6-month postoperative outcomes were comparable to those of the included patients, minimizing the risk for selection bias. All surgeries were uneventful, with successful implantation of the intraocular lenses within the capsular bag. No postoperative complications, including cystoid macular edema or intraocular pressure elevation, were detected.

The mean age at the time of the follow-up visit was 72.3 ± 6.7 years in the 6-month group and 77.4 ± 5.5 years in the 5-year group. Median ages were 72 years (range, 60–85) and 77 years (range, 65–89), respectively. In the 6-month group, there were 9 males and 11 females, while the 5-year group included 8 males and 10 females (Table 1).

### 3.1. PCO Development

The rate of PCO development and Nd:YAG laser capsulotomy was very low, ranging from 0% at 6 months to 5% (two eyes of two different patients) at 5 years. The two patients who developed visually significant PCO underwent Nd:YAG laser capsulotomy with visual improvement.

### 3.2. Visual Outcomes

The mean postoperative spherical equivalent (SE) was 0.33 ± 0.49 diopters (D) objectively and 0.05 ± 0.27 diopters (D) subjectively, with no statistically significant difference between the two measurements (*p* > 0.05); 100% of the eyes had a subjective SE within ±0.50 diopters (D).

All patients achieved high levels of monocular uncorrected and corrected distance (UDVA and CDVA), corrected intermediate (CIVA, 66 cm), and corrected near (CNVA, 40 cm) visual acuities. Table 2 presents visual outcomes at 6 months and 5 years postoperatively, reported separately for monocular and binocular measurements.

Binocular uncorrected visual acuity remained stable between the 6-month and 5-year follow-ups across all distances. Binocular uncorrected distance visual acuity (UDVA) showed no significant decline over time, with values of 0.03 ± 0.05 logMAR at 6 months and 0.04 ± 0.05 logMAR at 5 years (*p* = 0.358). Similarly, uncorrected intermediate visual acuity (UIVA) was 0.16 ± 0.10 logMAR at 6 months and 0.17 ± 0.05 logMAR at 5 years (*p* = 0.323). At 6 months, the mean uncorrected near visual acuity (UNVA) was 0.33 ± 0.05 logMAR, compared to 0.37 ± 0.05 logMAR at 5 years (*p* = 0.623).

**Figure 1:**
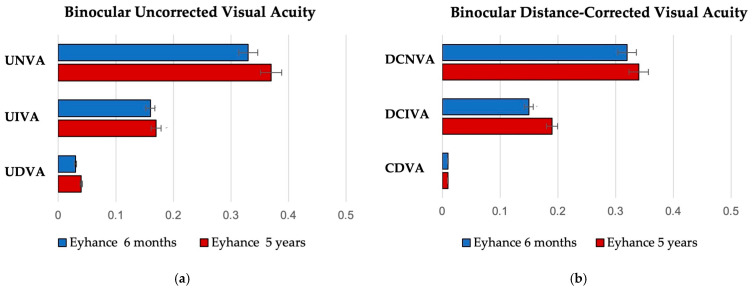
Postoperative binocular visual outcomes after 6 months and 5 years for both uncorrected (**a**) and distance-corrected (**b**) conditions (logMAR = logarithm of the minimum angle of resolution; UDVA = uncorrected distance visual acuity; UIVA = uncorrected intermediate visual acuity; UNVA = uncorrected near visual acuity; CDVA = corrected distance visual acuity; DCIVA = distance-corrected intermediate visual acuity; DCNVA = distance-corrected near visual acuity).

Binocular distance-corrected visual acuity also demonstrated long-term consistency, with no statistically significant differences observed in any subgroup. Corrected distance visual acuity (CDVA) remained excellent in both groups, with a mean of 0.01 logMAR (*p* = 0.748). Distance-corrected intermediate visual acuity (DCIVA) was 0.15 ± 0.08 logMAR and 0.19 ± 0.05 logMAR at the respective time points (*p* = 0.180). Distance-corrected near visual acuity (DCNVA) was 0.32 ± 0.04 logMAR at 6 months and 0.34 ± 0.07 logMAR at 5 years (*p* = 0.900). Binocular visual acuity results are shown in Figure 1.

**Figure 2:**
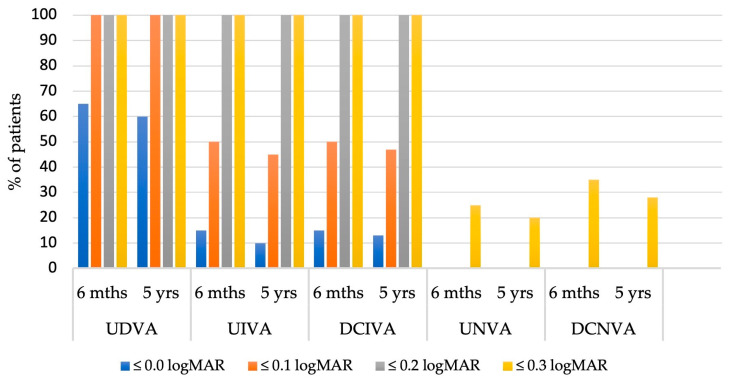
Distribution of binocular 6-month and 5-year postoperative visual outcomes (logMAR = logarithm of the minimum angle of resolution; UDVA = uncorrected distance visual acuity; UIVA = uncorrected intermediate visual acuity; DCIVA = distance-corrected intermediate visual acuity; UNVA = uncorrected near visual acuity; DCNVA = distance-corrected near visual acuity).

The distribution of binocular visual acuity (Figure 2) further supports the stability and efficacy of the Tecnis Eyhance IOL. In both groups, all patients achieved a binocular uncorrected distance visual acuity (UDVA) of ≤0.1 logMAR, while a UDVA of at least 0.0 logMAR was attained by 65% of patients at 6 months and 60% at 5 years postoperatively. Both uncorrected and distance-corrected intermediate visual acuities (UIVA and DCIVA) remained stable over time; approximately 45–50% of patients in both groups achieved an intermediate visual acuity (UIVA and DCIVA) of ≤0.1 logMAR, while all patients reached a visual acuity of ≤0.2 logMAR. Regarding uncorrected and distance-corrected near visual acuities (UNVA and DCNVA), between 20% and 35% of patients in both the 6-month and 5-year postoperative groups achieved a visual acuity of ≤0.3 logMAR, indicating a relatively limited but stable level of near vision outcome over time. These findings confirm that the Tecnis Eyhance IOL provides durable and effective visual performance from near to distance, with minimal decline over a 5-year period.

The spherical addition required for CIVA was slightly higher in the 5-year postoperative group compared to the 6-month group (1.21 ± 0.56 D and 1.15 ± 0.61 D, respectively; *p* = 0.187). Similarly, the spherical addition required for CNVA was also marginally greater in the 5-year group (2.44 ± 0.58 D) than in the 6-month group (2.31 ± 0.79 D), although the difference was not statistically significant (*p* = 0.223).

### 3.3. Binocular Defocus Curve

**Figure 3:**
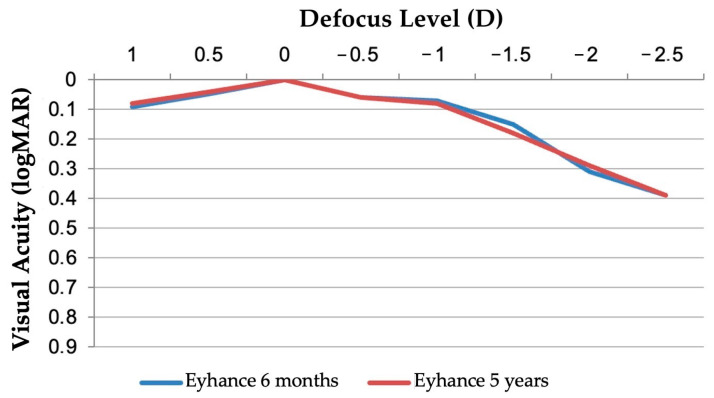
Mean defocus curves of the 6-month and the 5-year follow-up (logMAR = logarithm of the minimum angle of resolution).

The binocular distance-corrected defocus curve demonstrated sustained functional vision across a broad range of defocus levels in both the 6-month and 5-year follow-up groups (Figure 3). Peak visual acuity was observed at 0.00 D, corresponding to distance vision, with maintained acuity better than 0.2 logMAR between approximately −1.50 D and +0.50 D in both groups. This plateau confirms the Tecnis Eyhance IOL’s ability to provide extended depth of focus without compromising distance clarity. The 5-year curve closely paralleled the 6-month curve, indicating no significant decline in defocus performance over time.

### 3.4. Contrast Sensitivity

**Figure 4:**
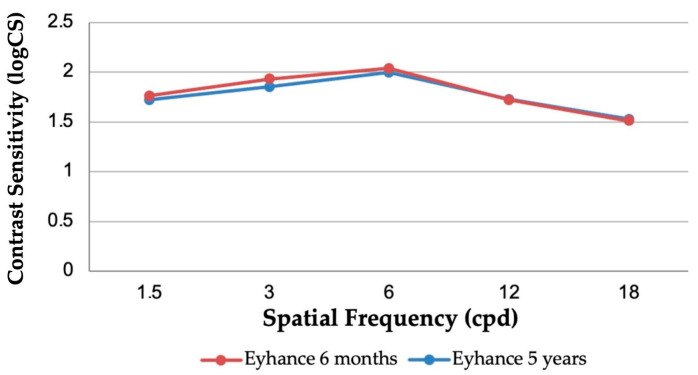
Contrast sensitivity measured under photopic conditions at different spatial frequencies (cycles per degree) at 6 months and 5 years postoperatively (logCS = log contrast sensitivity).

As shown in Figure 4, the mean photopic binocular contrast sensitivity remained stable over time, with similar performance observed between the 6-month and 5-year follow-up groups. At all tested spatial frequencies, values for the 5-year group closely mirrored those observed at 6 months (*p* > 0.05 for all comparisons), indicating preserved contrast sensitivity in the long term.

### 3.5. Optical Quality Parameters

Table 3 reports objective optical quality parameters, which remained stable between the 6-month and 5-year follow-up periods in patients implanted with the Tecnis Eyhance IOL. The mean objective scatter index (OSI) was slightly higher at 5 years (1.45 ± 0.51) compared to 6 months (1.36 ± 0.63), but the difference was not statistically significant (*p* = 0.139). Modulation transfer function (MTF) cutoff values were nearly identical (31.34 ± 4.02 c/deg at 5 years vs. 31.28 ± 8.70 c/deg at 6 months; *p* = 0.659), indicating consistent contrast resolution. The Strehl ratio also remained comparable (0.16 ± 0.04 at 5 years vs. 0.17 ± 0.04 at 6 months; *p* = 0.124), reflecting maintained optical quality and retinal image fidelity.

### 3.6. Halo Size

**Figure 5:**
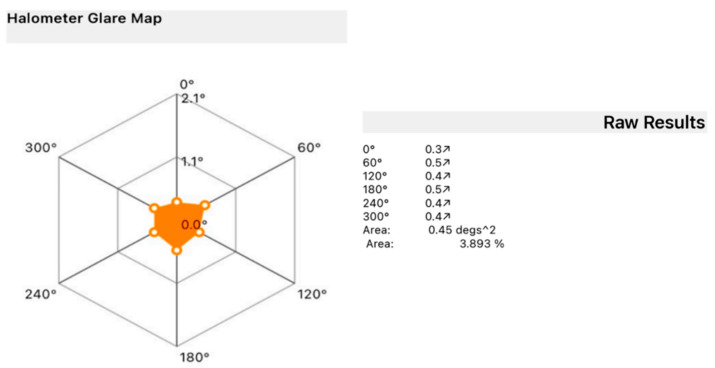
A result of a single examination with a halometer glare map and a numerical result indicating the corresponding area expressed in degrees^2^.

A halometer glare map was generated for each patient, monocularly (Figure 5). The distribution of average halo size at the 5-year follow-up was predominantly within a narrow range, as shown in Table 4. Most eyes (17 out of 36) reported halos between 0.51° and 0.6°, representing nearly half the sample. Smaller halo sizes were less common, with three eyes reporting halos in the 0.31–0.4° range and eight eyes in the 0.41–0.5° range. Moderate halo sizes of 0.61–0.8° were observed in seven eyes, while only one individual reported large halos exceeding 0.91°. These findings suggest a stable and relatively low clinical impact of photic phenomena over time.

### 3.7. Glare Perception and Spectacle Independence

No significant changes in the subjective perception of photic phenomena were observed over time. Scores derived from items 17 and 38 of the glare subscale of the NEI-RQL-42 questionnaire showed no significant difference between the 6-month and 5-year postoperative evaluations (*p* = 0.30), indicating stable patient-reported outcomes related to glare and halos.

**Figure 6:**
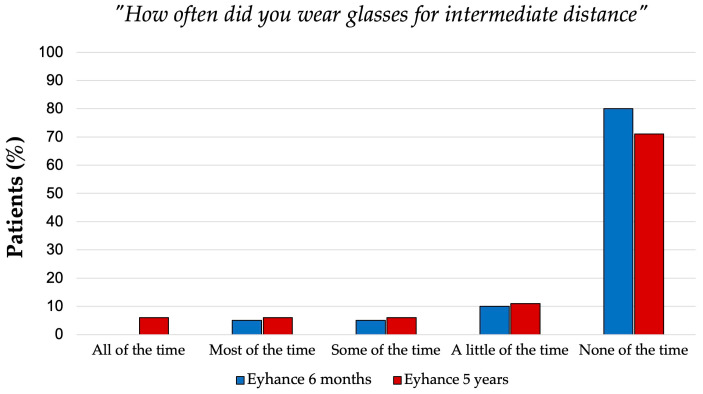
Distribution of patients’ answers to the question “How often did you wear glasses for intermediate distance?” of the Patient-Reported Spectacle Independence Questionnaire.

Spectacle dependence for distance and intermediate vision remained low at both follow-ups. These results were derived from the Patient-Reported Spectacle Independence Questionnaire (shown in Table 5), which evaluated the level of spectacle independence across different visual tasks. All patients (100%) were spectacle-free for distance vision, and more than 75% did not require glasses for intermediate tasks at both the 6-month and 5-year follow-ups. Near vision remained the most frequent reason for spectacle use in 95% of patients at 6 months, and 100% at 5 years required glasses for near tasks. When asked how often they wore glasses or contact lenses, 100% of patients at both 6 months and 5 years reported rarely or never needing them for distance vision. For intermediate tasks, 80% (6 months) and 71% (5 years) reported minimal or no reliance on corrective lenses (Figure 6). However, a higher level of dependence was noted for near tasks, with 40% or more of patients in both groups reporting frequent use of glasses. Moreover, at both time points, more than 50% of patients reported that they could only occasionally perform near-vision tasks comfortably without the use of glasses.

### 3.8. Effective Lens Position (ELP)

The effective lens position (ELP) of the Tecnis Eyhance IOL demonstrated long-term positional stability over the follow-up period. At 6 months postoperatively, the mean ELP was 4.08 ± 0.21 mm, and at 5 years, it was 4.15 ± 0.15 mm. As shown in Table 6, the difference between these time points was not statistically significant (*p* = 0.298), indicating that the IOL maintained a consistent axial position over time.

## 4. Discussion

As patient expectations following cataract surgery continue to rise, there is increasing demand for IOLs that enhance intermediate vision without inducing photic phenomena. Enhanced monofocal lenses such as the Tecnis Eyhance aim to address this need by extending depth of focus while preserving the optical quality typical of standard monofocal designs. Unlike most studies available in the literature, which predominantly focus on short- to medium-term follow-up periods (typically within 6 to 24 months), our study provides one of the most comprehensive 5-year evaluations of the Tecnis Eyhance IOL. This represents a significant contribution to the scientific literature, as it enables a reliable assessment of long-term refractive stability, optical quality, and patient satisfaction. It should be noted that the definition of “long-term” follow-up in the context of IOL outcomes remains inconsistent across studies, with some referring to durations as short as 24–30 months [12,13]. These durations, although informative, capture only a portion of the postoperative course.

Our study addresses this gap by providing robust and clinically meaningful data on the sustained efficacy and stability of the Tecnis Eyhance IOL up to 5 years after surgery. Our findings demonstrate that the visual and functional benefits observed at 6 months are largely maintained over time, underscoring the durability and reliability of this enhanced monofocal IOL.

### 4.1. Posterior Capsular Opacification and IOL Design Considerations

In addition to functional performance, the long-term success of an IOL also depends on its resistance to secondary complications such as posterior capsular opacification (PCO), which can compromise visual outcomes over time. PCO results from the migration and proliferation of residual lens epithelial cells (LECs) from the periphery of the capsular bag to the space between the intraocular lens (IOL) optic and the posterior capsule, leading to opacification [14]. This condition adversely affects optical clarity, visual quality, and function and typically manifests months to years after surgery. Reported incidence rates in adults range from 28% to 67%, reaching nearly 100% in children [15]. The biocompatibility of IOL materials significantly impacts PCO development. A meta-analysis of 11 studies involving 889 eyes demonstrated that hydrophobic IOLs exhibit lower subjective PCO scores compared to hydrophilic lenses. Hydrophobic materials with sharp-edged designs inhibit macrophage and LEC adhesion, reducing PCO formation. In contrast, hydrophilic polymers and silicone materials tend to promote fibrosis, cell migration, and proliferation, increasing PCO rates [16].

In our study, the incidence of posterior capsular opacification (PCO) was notably low, with no cases observed at 6 months postoperatively and a 5% rate at 5 years. Only two eyes (from two patients) developed clinically significant PCO requiring Nd:YAG laser capsulotomy during the entire follow-up period. Despite the relatively small sample size, this low rate is clinically meaningful and suggests sustained posterior capsular clarity over time. These findings are in line with previous studies highlighting the impact of IOL design on PCO prevention. In particular, Mencucci et al. [17] conducted a comparative evaluation of hydrophobic acrylic IOLs and demonstrated that lenses with a continuous 360° sharp posterior optic edge, such as those in the Tecnis platform, significantly reduce the incidence of PCO and the need for Nd:YAG laser capsulotomy. The sharp edge acts as an effective mechanical barrier, limiting the migration of lens epithelial cells (LECs) on the posterior capsule.

This low incidence of PCO likely contributed to the maintenance of high-quality visual outcomes over time, minimizing secondary visual degradation and supporting the overall optical stability of the IOL.

### 4.2. Long-Term Visual and Optical Performance

Visual acuity outcomes remained stable over the 5-year period, with no significant differences in uncorrected or corrected visual acuity at near, intermediate, or distance when compared to the 6-month results. These findings are consistent with previous studies suggesting that the Tecnis Eyhance IOL may offer improvements in intermediate vision compared to standard monofocal lenses while preserving distance visual acuity [18]. The extended depth of focus provided by the aspheric anterior surface of the Tecnis Eyhance optic is likely responsible for the functional gains in intermediate vision, a well-known limitation in traditional monofocal IOLs [19,20].

The binocular defocus curve results further support the lens’s long-term functional performance, confirming maintained intermediate visual acuity over a range of defocus levels. Importantly, this enhancement does not appear to compromise distance vision or increase the prevalence of photic phenomena, often observed in multifocal or extended depth-of-focus (EDOF) IOL designs [21,22].

Our findings also support the preservation of contrast sensitivity over time. Figure 4 illustrates the similarity in photopic contrast sensitivity between the 6-month and 5-year groups, with no statistically significant differences at any spatial frequency. This suggests sustained visual quality and neural contrast processing over a 5-year period, a reassuring indicator of long-term retinal image quality.

Objective measures of optical quality—including the objective scatter index (OSI), modulation transfer function (MTF) cutoff, and Strehl ratio—remained stable from 6 months to 5 years postoperatively, confirming long-term consistency in optical performance.

### 4.3. Effective Lens Position (ELP) Stability

The stability of the effective lens position (ELP) is a key determinant of long-term refractive predictability and optical performance in pseudophakic eyes. Shifts in ELP can significantly affect postoperative refraction, especially with modern IOLs designed to enhance intermediate or near vision [23,24].

In our 5-year follow-up, no clinically significant changes in refractive error were observed, indicating excellent ELP stability over time. Additionally, imaging assessments revealed no evidence of axial IOL movement, tilt, or decentration, supporting the mechanical stability of the Tecnis Eyhance IOL within the capsular bag. The open C-loop haptic configuration typical of Tecnis IOLs, including the Eyhance, can guarantee a reliable capsular bag engagement. A swept-source AS-OCT study [25] comparing plate-haptic, closed C-loop, and standard C-loop designs reported a slight increase in mean decentration and tilt for C-loop lenses, although these remained within clinically acceptable limits [26]. Moreover, the hydrophobic acrylic material used in the Tecnis platform is known to promote strong early postoperative capsular fixation through fibronectin-mediated adhesion, which stabilizes the IOL position and minimizes movement over time. Previous studies have shown that hydrophobic acrylic lenses with a stable haptic design, such as those in the Tecnis platform, offer superior positional stability due to their material properties and design geometry [27]. This positional consistency contributes to reliable refractive outcomes and helps to preserve the IOL’s intended optical performance over extended periods.

### 4.4. Patient-Reported Outcomes and Spectacle Independence

Patient satisfaction and functional independence are critical indicators of success following IOL implantation. Patient-reported data from the Spectacle Independence Questionnaire revealed consistent and meaningful levels of spectacle independence, particularly for distance and intermediate vision. These findings suggest that the Tecnis Eyhance IOL provides sustained spectacle independence for distance and intermediate vision, with near correction remaining the primary area of residual dependency.

The evaluation of photic phenomena showed that halo size remained in the mild-to-moderate range for most patients, with the majority reporting halo diameters between 0.51° and 0.6°, and only a small subset exceeding 0.8°. This favorable profile contrasts with that of many multifocal and EDOF lenses, where dysphotopsias can significantly impact quality of life and patient satisfaction [28]. Unfortunately, as mentioned before, objective measurement of halo size was performed only at the 5-year follow-up, preventing a longitudinal comparison of halo size progression. However, subjective assessment of glare and halo perception using the NEI-RQL-42 questionnaire was conducted at both time points and showed no significant differences between 6 months and 5 years, suggesting overall stability in patient-reported photic symptoms over time.

### 4.5. Study Limitations

We acknowledge several limitations of our study. First, the sample size is relatively small and selective, which may limit the generalizability of the findings. In fact, the study cohort was carefully selected using stringent inclusion and exclusion criteria to minimize confounding factors and ensure uniformity. While this homogeneity helped minimize potential confounding variables, it may limit the applicability of the results to the general cataract population seen in everyday clinical settings. Although the study achieved a high follow-up rate (90%), the exclusion of two patients due to age-related macular degeneration slightly reduced the final sample and may have introduced selection bias if the excluded patients differed systematically from those who remained. However, the baseline characteristics and 6-month outcomes of these excluded patients did not differ significantly from the rest of the cohort, suggesting minimal impact on the overall findings.

Second, no comparative arm with other IOL types, such as standard monofocal or EDOF lenses, was included, preventing a direct comparison of long-term outcomes; nonetheless, long-term results for monofocal lenses have been reported and validated in prior studies. However, the primary objective of this study was not to compare different IOL designs but rather to provide a long-term longitudinal assessment of the Tecnis Eyhance IOL’s optical stability, visual performance, and patient satisfaction.

Third, although the absence of age-matched controls may introduce potential confounding from natural aging, the stability of visual acuity, contrast sensitivity, and optical quality over 5 years supports that the outcomes are primarily related to the IOL’s performance.

Moreover, although contrast sensitivity was assessed under photopic conditions, the study did not include specific functional task-based evaluations of intermediate vision.

Furthermore, another limitation concerns the halometry evaluation, which was performed only at the 5-year follow-up due to the device being unavailable at the 6-month visit. Consequently, longitudinal comparison of objectively measured halo size was not possible, limiting the interpretation of photic phenomena progression.

Future prospective multicenter studies with larger and more heterogeneous populations, as well as randomized comparisons and multiple-surgeon studies, are warranted to confirm and extend these findings.

## 5. Conclusions

Nowadays, cataract surgery is increasingly considered a refractive procedure, often recommended for patients with a long life expectancy and the need to preserve clear vision for many years without contrast sensitivity loss and with an extended depth of focus. Therefore, our purpose stems from the interest in determining whether the visual advantages provided by the Tecnis Eyhance at 6 months can be maintained over time, despite the natural aging processes affecting ocular and neurobiological tissues. In conclusion, the 5-year follow-up data confirm the sustained performance of the Tecnis Eyhance IOL, with stable optical and visual outcomes, preserved contrast sensitivity, minimal dysphotopsia, and high levels of spectacle independence for distance and intermediate tasks. These results support the Tecnis Eyhance IOL as a reliable long-term solution for patients seeking a monofocal lens with enhanced intermediate performance and minimal compromise on visual quality.

## Figures and Tables

**Table 1:** Demographic characteristics of the included patients.

Parameter	Eyhance 6 Months	Eyhance 5 Years
**Number of patients (eyes)**	20 (40)	18 (36)
**Age (years)**		
*Mean ± SD*	72.3 ± 6.7	77.4 ± 5.5
*Median (range)*	72 (60–85)	77 (65–89)
**Sex**		
*Male*	9	8
*Female*	11	10
**Follow-up time (years)**	0.5 ± 0.1	5.6 ± 0.4

**Table 2:** Monocular and binocular visual outcome.

	6-Month Follow-Up	5-Year Follow-Up	*p* Value
**UDVA monocular**			
Mean ± SD	0.04 ± 0.05	0.06 ± 0.05	0.188
**CDVA monocular**			
Mean ± SD	0.02 ± 0.04	0.03 ± 0.09	0.447
**UDVA binocular**			
Mean ± SD	0.03 ± 0.05	0.04 ± 0.05	0.358
**CDVA binocular**			
Mean ± SD	0.01 ± 0.04	0.01 ± 0.01	0.748
**UIVA monocular**			
Mean ± SD	0.28 ± 0.11	0.28 ± 0.10	0.534
**DCIVA monocular**			
Mean ± SD	0.27 ± 0.11	0.25 ± 0.09	0.819
**CIVA monocular**			
Mean ± SD	0.06 ± 0.09	0.07 ± 0.08	0.384
**UIVA binocular**			
Mean ± SD	0.16 ± 0.10	0.17 ± 0.05	0.323
**DCIVA binocular**			
Mean ± SD	0.15 ± 0.08	0.19 ± 0.05	0.180
**CIVA binocular**			
Mean ± SD	0.04 ± 0.05	0.06 ± 0.05	0.483
**UNVA monocular**			
Mean ± SD	0.46 ± 0.13	0.48 ± 0.14	0.630
**DCNVA monocular**			
Mean ± SD	0.44 ± 0.05	0.44 ± 0.14	0.840
**CNVA monocular**			
Mean ± SD	0.06 ± 0.08	0.05 ± 0.07	0.770
**UNVA binocular**			
Mean ± SD	0.33 ± 0.05	0.37 ± 0.05	0.623
**DCNVA binocular**			
Mean ± SD	0.32 ± 0.04	0.34 ± 0.07	0.900
**CNVA binocular**			
Mean ± SD	0.03 ± 0.05	0.02 ± 0.04	0.867

**Table 3:** Optical quality parameters analyzed using a double-pass system at a 4.0 mm pupil diameter (OSI = objective scatter index; MTF = modulation transfer function).

	Eyhance 6 Months	Eyhance 5 Years	*p* Value
OSI	1.36 ± 0.63	1.45 ± 0.51	0.139
MTF cutoff (c/deg)	31.28 ± 8.70	31.34 ± 4.02	0.659
Strehl ratio	0.17 ± 0.04	0.16 ± 0.04	0.124

**Table 4:** Distribution of average monocular halo sizes measured at the 5-year follow-up.

Average Halo Size (Degrees^2^)	Numbers of Eyes (n)
0.31–0.4	3
0.41–0.5	8
0.51–0.6	17
0.61–0.7	5
0.71–0.8	2
0.81–0.9	-
>0.91	1

**Table 5:** Patient-Reported Spectacle Independence Questionnaire results.

		Eyhance 6 Months n (%)					Eyhance 5 Years n (%)				
		Cat. 1	Cat 2.	Cat. 3	Cat. 4	Cat. 5	Cat. 1	Cat. 2	Cat. 3	Cat. 4	Cat. 5
**Did you need glasses for:**	Distance	-	20 (100%)	-	-	-	-	18 (100%)	-	-	-
	Intermediate	4 (20%)	16 (80%)	-	-	-	4 (22%)	14 (78%)	-	-	-
	Near	19 (95%)	1 (5%)	-	-	-	18 (100%)	-	-	-	-
**How often did you wear glasses or contacts for:**	Distance	-	-	-	-	20 (100%)	-	-	-	-	18 (100%)
	Intermediate	-	1 (5%)	1 (5%)	2 (10%)	16 (80%)	1 (6%)	1 (6%)	1 (6%)	2 (11%)	13 (71%)
	Near	8 (40%)	4 (20%)	4 (20%)	2 (10%)	2 (10%)	8 (45%)	2 (11%)	2 (11%)	4 (22%)	2 (11%)
**Were you able to function comfortably** **without glasses or contacts for:**	Distance	20 (100%)	-	-	-	-	18 (100%)	-	-	-	-
	Intermediate	12 (60%)	4 (20%)	2 (10%)	2 (10%)	-	14 (78%)	-	4 (22%)	-	-
	Near	-	1 (5%)	3 (15%)	4 (20%)	12 (60%)	-	2 (11%)	5 (28%)	9 (50%)	2 (11%)

**Table 6:** ELP for Tecnis Eyhance at 6 months and 5 years postoperatively. Results are expressed as mean ± standard deviation. (ELP = effective lens position.)

	6 Months	5 Years	*p* Value
ELP (mm)	4.08 ± 0.21	4.15 ± 0.15	0.298

## Data Availability

The raw data supporting the conclusions of this article will be made available by the authors on request.
